# Estimating presenteeism from repeated smartphone-based multimodal behavioral responses

**DOI:** 10.1371/journal.pone.0354342

**Published:** 2026-09-16

**Authors:** Taiga Noguchi, Shotaro Doki, Masakazu Hirokawa, Soma Nishimura, Katsuya Hotta, Yuya Iwata, Naoko Kouda, Shota Matsumoto, Kenji Suzuki

**Affiliations:** 1 Graduate School of Science and Technology, University of Tsukuba, Tsukuba, Ibaraki, Japan; 2 Institute of Medicine, University of Tsukuba, Tsukuba, Ibaraki, Japan; 3 Minds in Co., Ltd., Chuo-ku, Tokyo, Japan; 4 Data Science Laboratories, NEC Corporation, Meguro-ku, Tokyo, Japan; 5 Center for Cybernics Research, University of Tsukuba, Tsukuba, Ibaraki, Japan; 6 Graduate School of Comprehensive Human Sciences, University of Tsukuba, Tsukuba, Ibaraki, Japan; 7 Retail & Consumer Service Division, Sojitz Corporation, Chiyoda-ku, Tokyo, Japan; 8 Human Capital Dept. 2, Sojitz Corporation, Chiyoda-ku, Tokyo, Japan; 9 Public Relations Dept., Sojitz Corporation, Chiyoda-ku, Tokyo, Japan; 10 Digital Business Division, DG COMMUNICATIONS Co., Ltd., Shinjuku-ku, Tokyo, Japan; 11 Institute of Systems and Information Engineering, University of Tsukuba, Tsukuba, Ibaraki, Japan; University of Lahore - Raiwind Road Campus: The University of Lahore, PAKISTAN

## Abstract

Presenteeism, attending work despite physical or mental health problems, is a major source of productivity loss worldwide, yet its early detection in everyday settings remains challenging. Conventional self-report instruments are time-consuming and require psychiatrist interpretation, which limits their scalability. We developed a multimodal machine-learning model that estimates work-function impairment as an indicator of presenteeism from brief smartphone-based dialogues. 38 employees recorded short self-report videos (10–30 s) using front-facing smartphone cameras, which were independently rated on an ordinal three-level work-function scale (Healthy / Moderate / Unwell) by three psychiatrists; the consensus label served as supervision. We tested whether integrating multimodal behavioural signals (acoustic, facial, and linguistic) would provide additional cues beyond linguistic content alone, particularly when verbal cues are sparse. Acoustic, facial, and linguistic features were extracted from each clip and integrated using a three-stream Attention-based Multiple Instance Learning (Attention-MIL) framework with learned late fusion, an ordinal-regression head, and class-prior logit adjustment. Generalisation was assessed under a participant-disjoint protocol (25-fold repeated GroupKFold; *n* = 1,768 out-of-fold clips from 29 participants). On the Full configuration, the proposed multimodal framework achieved macro-F1 = 0.772 (95% CI [0.697, 0.817]), accuracy = 0.840, macro-AUROC = 0.940, and low expected calibration error (ECE ≈0.024). At the aggregate level, the four text-containing configurations (Full / Audio+Text / Face+Text / Text-only) were mutually indistinguishable on macro-F1 (Holm–Bonferroni *p*_adj_ > 0.40), whereas text-free configurations performed substantially worse (Δ<−0.33, *p*_adj_ < 0.001), confirming the necessity of the linguistic channel. In an exploratory subgroup analysis, Audio+Text outperformed Text-only in the clinically ambiguous subgroup—short-speech responses from non-Healthy participants (*n* = 77; Δ macro-F1 =+0.030, exploratory, requires replication). These findings provide localised but clinically meaningful support for the multimodal hypothesis under low-information conditions and motivate prospective replication.

## Introduction

### Burden and public-health significance

Presenteeism, broadly defined as attending work despite physical or mental health problems, is increasingly viewed as a major contributor to productivity loss and diminished well-being. Early empirical work suggested that working while ill is common in working populations and is shaped by occupational conditions (e.g., replaceability and job demands) in addition to health status [1]. Building on this, later syntheses emphasize that presenteeism is not only prevalent but also consequential. A longitudinal review of sickness presenteeism reports that repeated or sustained presenteeism is associated with adverse health and well-being over time, indicating that it is more than a short-term coping behavior [2]. Accordingly, reviews and critical overviews position mental health as a central upstream factor for workplace productivity and highlight that the productivity burden is mediated through both absenteeism and presenteeism pathways [3,4].

Although the burden is observed internationally, its magnitude and expression depend on local measurement practices and organizational context. For example, studies in both workplace and clinical settings quantify productivity loss by jointly considering absenteeism and presenteeism, reinforcing that the key outcome is functional impact rather than symptoms alone [5,6]. In Japan, employer- and cohort-based analyses further indicate that productivity loss attributable to presenteeism can account for a substantial share of total health-related costs [7–9]. Longitudinal evidence also suggests that psychological distress can be followed by persistent productivity impairment, implying that missed early signals may translate into prolonged functional loss [10,11]. These findings motivate scalable, low-burden approaches for early identification of work-function impairment in occupational settings.

### Limitations of current detection and monitoring approaches

Existing detection and monitoring approaches largely rely on self-report instruments, periodic screenings, and occasional specialist assessments. While useful, these approaches have three persistent limitations in real-workplace deployment: (i) bias and introspective limits in self-report, (ii) high burden and low temporal resolution, and (iii) construct heterogeneity and context dependence.

First, self-reports can systematically underestimate strain. Organizational research suggests that employees may defensively downplay stress and strain, implying divergence between reported states and actual burden even when an instrument is well designed [12]. More broadly, self-report presupposes accurate introspection, yet perceived “being able to work” may not align with externally observable functional impairment.

Second, questionnaires and screenings impose burden and therefore cannot be deployed at high frequency. In practice, completing multi-item instruments can be time-consuming and disruptive, making daily or near-daily administration unrealistic in many workplaces. Consequently, periodic screening tends to capture only coarse changes and can miss shorter-term within-person dynamics of work limitation and functioning [13]. Specialist-led assessments provide richer judgment but are costly and difficult to maintain routinely at scale.

Third, heterogeneity in measurement and definition complicates both inference and implementation. Two major constructs—“sickness presenteeism” (working despite illness) and “impaired work function” (reduced functional capacity while at work)—are related but not equivalent, and estimates based on them are not interchangeable [14]. Moreover, organizational context can reshape both reporting and attendance behavior, which can alter the validity of self-report and presence-based indicators. Structural factors such as job flexibility and job security further affect mental-health outcomes and work attendance, limiting one-size-fits-all monitoring schemes [15]. This conceptual non-equivalence complicates benchmarking, label design, and the interpretation of predictive models, particularly when models are trained on symptom proxies rather than direct assessments of functioning during work.

Taken together, these limitations argue for objective, low-burden, and higher-frequency indicators that complement questionnaires and periodic assessments. Such indicators should be feasible in naturalistic work settings and sufficiently interpretable to support screening and initial response decisions in occupational health.

### Advances in digital and multimodal AI for work-function assessment

Digital phenotyping and multimodal machine learning (MML) provide a technical foundation for translating everyday behavioral signals into quantitative indicators. Single-modality approaches (e.g., speech or facial cues) can capture mental-state–related variation, but they are vulnerable to missingness and context in short, everyday recordings. Multimodal fusion can integrate complementary cues and stabilize inference under such constraints [16,17]. At the deployment layer, smartphone-based sensing studies illustrate low-burden designs that can be embedded into daily routines [18,19].

However, prior multimodal studies have primarily targeted proximal labels such as affective states, stress markers, or questionnaire-derived severity scores [16,17,20,21], which are not direct assessments of work functioning during active employment. This leaves a methodological disconnect between advances in multimodal behavioral modeling and psychiatrist-consensus–grounded assessment of work-function impairment.

### Study aim and methodological contributions

Motivated by this methodological gap, we develop and evaluate a psychiatrist-consensus–anchored multimodal model that estimates ordinal work-function impairment, interpreted here as a functional manifestation of presenteeism in occupational settings, from brief smartphone-based dialogues. Rather than predicting affective states or questionnaire scores, the proposed model is directly supervised by psychiatrist consensus ratings of functional impairment during work.

The model integrates linguistic content with nonverbal behavioral cues, including vocal prosody, facial signals, and head-motion dynamics, and is designed for short interactions (10–30 s) and low-burden capture in naturalistic workplace settings. Conceptually, multimodal behavioral signals captured by smartphones are treated as a low-burden statistical approximation of psychiatrist judgment, not as a replacement for clinical assessment.

Our contributions are threefold. First, we show that brief smartphone-recorded dialogues contain sufficient multimodal information to approximate psychiatrist consensus ratings of work-function impairment. Second, by explicitly modeling ordinal severity, the proposed approach preserves graded transitions between functional states, enabling more fine-grained assessment than nominal classification. Third, we analyze modality contributions and disagreement cases to clarify how nonverbal cues refine decision boundaries in conjunction with language.

## Materials and methods

### Study design and data collection

**Fig 1:**
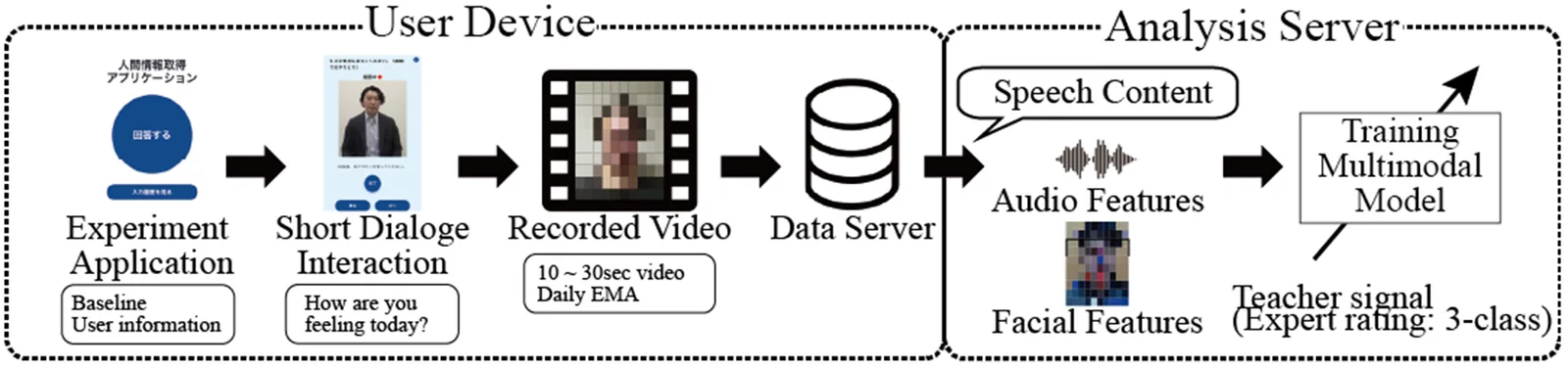
Overview of the study workflow. Participants responded to short video prompts and EMA items via a custom iOS application in daily work settings. Data were uploaded through encrypted channels to a study server and processed offline to extract multimodal behavioral signals and train the proposed models. Screenshots show the application interface as implemented in the study.

This prospective observational study employed a custom iOS application installed on company-issued iPhones to collect short self-report videos (front camera; face and speech) and ecological momentary assessments (EMA) in naturalistic work settings. Fig 1 provides an end-to-end overview of the data-collection and analysis workflow, including on-device prompting, secure upload, and offline processing for model development.

At first launch, participants completed a one-time baseline questionnaire covering demographics, personality, sleep, and psychological distress (S5 Table). At each prompt thereafter, participants positioned their face within an on-screen frame, recorded a 10–30 s video reply to “How are you feeling today?”, and answered brief EMA items (S6 Table). All responses were transmitted via encrypted channels to a study server for storage and preprocessing. Collected media were subsequently processed offline to extract facial, acoustic, and linguistic features and to train and evaluate multimodal models (Fig 1).

Daily sampling followed standard EMA practice [22]. Affect was recorded using Russell’s circumplex coordinates (valence×arousal) [23,24] and is summarized descriptively in S6 Table. Baseline psychometric scales and daily EMA items were collected to support contextual understanding during psychiatrist consensus labeling and for descriptive characterization of the cohort (S5 and S6 Tables). They were not incorporated as input features in the machine-learning models.

Participants were white-collar employees from a single company (enrolled $n_{participants}=39$, age 20–60 years), including 13 women. Of these, $n_{participants}=38$ provided valid video submissions (mean age = 41.4 years); the subset entering the supervised analysis (after feature-extraction and labeling availability, detailed in *Feature extraction and preprocessing*) was $n_{participants}=34$. Participation was voluntary, with written informed consent obtained from all participants. Data were collected from February 15 to May 20, 2024 (approximately 3 months), during which participants were asked to submit at least two responses per day (pre- and post-work). The protocol was approved by the Institutional Review Board of the University of Tsukuba (Approval No. 1662−6). Cohort composition and engagement are summarized descriptively in the Results section.

### Baseline and ecological momentary assessments

At study entry, participants completed a baseline questionnaire comprising demographics and three validated psychometric scales: TIPI/TIPI-J [25,26], AIS [27,28], and K6 [29]; full items and response formats are in S5 Table. During the study period, daily ecological momentary assessments (EMAs) captured location, work status, previous-night sleep, affect on Russell’s circumplex [23,24], workload factors, self-rated job performance, and working hours (full items in S6 Table). Both the baseline questionnaire and the EMA were used to characterise the cohort descriptively and to provide contextual reference information to psychiatrists during labeling; they were not used as predictive input features in the multimodal models.

### Psychiatrist consensus labeling

Each valid video clip was independently reviewed by three psychiatrist raters together with the corresponding EMA responses. Raters assigned one of three ordinal labels representing work-function impairment, which we interpret as an indicator of presenteeism: 0 = Healthy, 1 = Moderate, and 2 = Unwell. To reduce ambiguity and enhance consistency, raters were instructed to base their judgments primarily on observable behavior in the video, including facial expressions, speech characteristics, and spoken content. Information from baseline questionnaires and EMA responses was used only as secondary contextual cues to support interpretation when needed.

All three raters were board-certified psychiatrists with occupational mental health expertise (one Designated Mental Health Physician with additional social-medicine credentials; two certified occupational physicians).

A single psychiatrist consensus label was obtained for each clip by majority vote across the three ratings. Clips showing complete dispersion (i.e., one rater assigned each of the three classes—Healthy, Moderate, and Unwell) were regarded as ambiguous and excluded from both model training and evaluation; this criterion removed 10 clips (≈0.56% of the 1,791 labeling-eligible clips), leaving 1,781 labeled clips before the participant-level inclusion criterion described in *Feature extraction and preprocessing* was applied.

Inter-rater agreement across the three psychiatrists was substantial (Fleiss’ κ=0.693; pairwise Cohen’s κ=0.642 [R1–R2], 0.819 [R1–R3], 0.617 [R2–R3]; raw agreement 81.5%/90.5%/80.0%), supporting the validity of aggregating individual ratings via majority vote. Accordingly, the majority-vote label was adopted as the ordinal target for supervised learning. The structure of inter-rater disagreement and its implications for per-class performance are detailed in the Limitations section.

The overall labeling protocol builds on our prior response-based assessment framework for camera-mediated self-reports [19]. Because the endpoint in the present study is a psychiatrist consensus ratings of work-function impairment rather than an affective proxy or questionnaire score, the supervisory signal encodes clinically grounded judgments about functional status during everyday work. Accordingly, the proposed models are trained to capture statistical regularities that align with psychiatrist reasoning in naturalistic occupational contexts.

### Feature extraction and preprocessing

To operationalize psychiatrist-labeled work-function impairment from observable behavior, we extracted multimodal behavioral representations from each valid video. Specifically, three primary feature streams were derived to capture complementary aspects of facial expression, speech acoustics, and linguistic content.

Facial features were obtained via OpenFace and included facial action units, head pose, and gaze [30]. Acoustic features were computed using a Praat-compatible paralinguistic pipeline and comprised fundamental frequency (F0), intensity, harmonics-to-noise ratio, formants, spectral measures, and MFCCs [31]. Linguistic features were derived by transcribing speech with Whisper [32] and embedding utterances using transformer encoders from the Sentence-BERT family [33,34].

To integrate these heterogeneous streams at the temporal level, Whisper timestamps were used to define utterance boundaries. Text embeddings and turn identifiers were broadcast across their corresponding time spans to align linguistic information with continuous facial and acoustic signals. Facial and acoustic streams were temporally aligned, resampled to 100 Hz, and aggregated into 50 ms frames with 50 ms hops (20 Hz).

**Fig 2:**
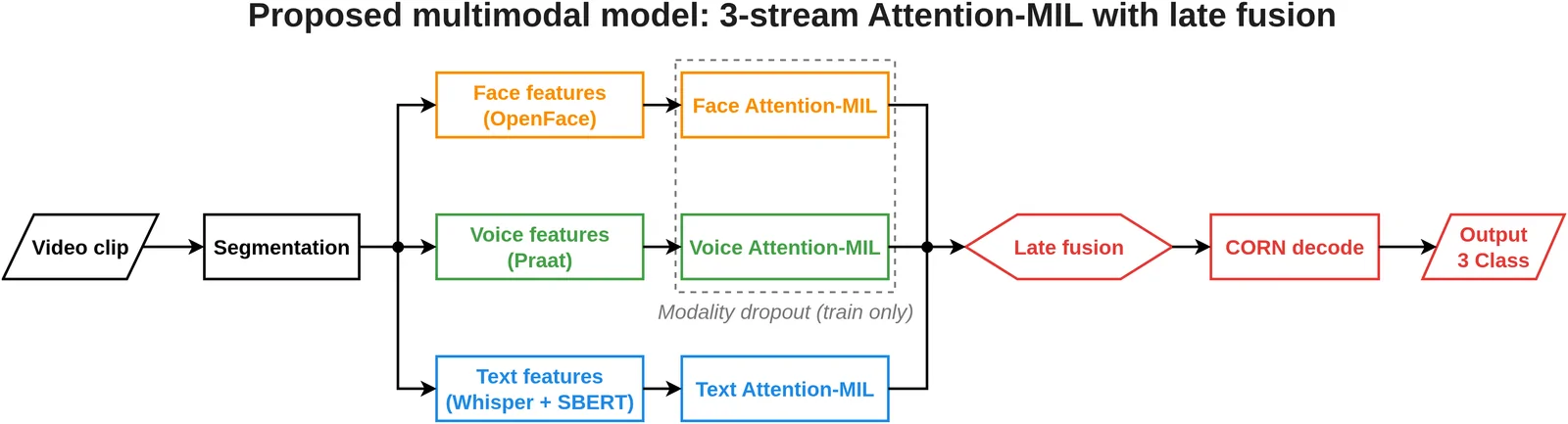
Proposed multimodal modeling pipeline. Each smartphone-dialogue clip is first segmented into speech segments, from which three parallel feature streams (face, voice, text) are extracted and independently encoded by per-stream Attention-MIL blocks. The three stream-level representations are combined by late fusion with logit adjustment and decoded into the three ordinal classes (Healthy / Moderate / Unwell) by a CORN ordinal head. Modality dropout on the face and voice streams is applied during training only. Implementation details (feature dimensions, time alignment, fusion equation, CORN decoding, and training loss) are described in Methods, *Feature extraction and preprocessing* and *Model and evaluation*.

A limited set of survey-derived attributes was retained only to support normalization procedures and stratified descriptive summaries. All normalization statistics were computed exclusively within each training fold and applied to the corresponding held-out fold, thereby preventing information leakage across participants. The resulting aligned multimodal sequences served as input to the modeling pipeline described below and illustrated in Fig 2.

#### Participant- and clip-level inclusion criteria.

Of the 38 participants with valid video submissions, 34 contributed at least one clip with successful three-modality feature extraction (facial action units, vocal acoustics, and automatic transcription); the remaining 4 participants had no clip for which automatic speech recognition produced a usable transcript, despite valid face and voice signals, and were therefore not carried forward into the analysis set. The 1,791 labeling-eligible clips from these 34 participants are the input to the psychiatrist labeling step described above.

Because our evaluation design is participant-disjoint cross-validation, each held-out individual must contribute a longitudinal sample large enough to estimate transfer to unseen people; participants with only a few days of recording—and consequently few clips and limited label variability—cannot serve this role. We therefore restricted analyses to participants with at least 15 days between their first and last recording. Five short-span participants were excluded (≤14 days of recording activity); together they contributed 13 clips (≈0.7% of the available data). The final analysis set comprises *n* = 1,768 clips from 29 participants (overall participant flow: 38 valid submissions → 34 with three-modality features and psychiatrist labels → 29 after short-span exclusion; overall clip flow: 1,791 labeling-eligible clips → 1,781 after consensus-dispersion filtering → 1,768 after short-span participant exclusion).

### Model and evaluation

We trained a three-stream Attention-MIL model [35] that maps temporally aligned facial, acoustic, and linguistic feature sequences (${𝐗}_{f},{𝐗}_{v},{𝐗}_{t}$) to a probability over the three ordinal classes (Fig 2). The per-modality encoders are attention-pooled and combined via learnable, simplex-constrained late fusion before being passed through a rank-consistent ordinal head:


$$\begin{array}{c} \hat{p}(y|{𝐗}_{f},{𝐗}_{v},{𝐗}_{t})\;=\;CORN\;(\;\sum_{m\in \{f,v,t\}}w_{m}\;\phi_{m}({𝐗}_{m})), \end{array}$$ (1)

where $\phi_{m}$ is a per-modality attention-pooled encoder [35], $\left\{w_{m}\right\}$ are simplex-constrained learnable fusion weights, and CORN(·) is the rank-consistent ordinal head of [36] with logit adjustment (τ=1.0) [37] for class imbalance. The network is trained against a fused-logit ordinal loss with an auxiliary text-stream regulariser:


$$\begin{array}{c} ℒ\;=\;{ℒ}_{CORN+LA}(z_{fuse},y)\;+\;\lambda_{aux}\;{ℒ}_{CORAL}(z_{t},y), \end{array}$$ (2)

where $z_{fuse}$ is the fused logit, $z_{t}$ is the text-stream logit, and $\lambda_{aux}=0.2$ weights an auxiliary CORAL-style ordinal BCE [38]. During training, modality dropout [39] independently masks the face and voice streams with $p_{f}=p_{v}=0.3$. Optimisation uses AdamW (weight decay 10^−4^); hyperparameters are selected per outer fold by Optuna Bayesian optimisation [40] (TPE sampler, 10 trials). Full implementation details are released alongside the code.

To evaluate generalization performance while preventing identity leakage, we employed 25-fold repeated participant-disjoint cross-validation (5 repeats × 5-fold GroupKFold), assigning all clips from the same participant to a single fold. All out-of-fold (OOF) predictions across all 25 folds ($n_{oof\_clips}=1,768$) were aggregated as the primary evaluation set. OOF predictions were used for all reported classification, discrimination, and calibration metrics. For modality ablation comparisons, fold-level metric vectors (*n* = 25) were used for paired *t*-tests with *df* = 24; since the 5×5 repeated structure makes *t*he 25 folds non-independent, single-configuration uncertainty is additionally quantified by the participant-level cluster bootstrap described below. For subgroup analyses with limited stratum size, fold-level paired *t*-tests are restricted to folds whose test partition contains (i) at leas*t* 5 stratum clips and (ii) more than one true class. This pre-specified criterion depends only on the test partition’s clip count and class membership, not on any model’s predictions, so it cannot preferentially favour any configuration; small-sample F1 is otherwise unstable (a single misclassification on fewer than 5 clips shifts macro-F1 by 0.2–0.3) or mathematically uninformative (single-class macro-F1 collapses to one per-class term). A sensitivity analysis replacing excluded folds with Δ=0 and re-testing on the full 25-fold set is reported alongside the primary result in Results. Metric uncertainty was quantified by a participant-level (cluster) bootstrap with *B* = 2,000 resamples of the 29 participants, yielding 95% percentile confidence intervals. Holm–Bonferroni correction [41] was applied across the family of six paired modality contrasts against the Text-only baseline to control the family-wise error rate. Throughout this paper, we use the phrase *statistically indistinguishable* to mean “no detectable difference at α=0.05 after Holm–Bonferroni correction”; this is a non-rejection of the null and does not constitute formal equivalence in the sense of a TOST procedure with a pre-specified margin. Subgroup-level contrasts (Results, *Subgroup analysis: where nonverbal modalities contribute*) are reported uncorrected and explicitly labelled as exploratory; the multiplicity caveat for the speech-duration × clinical-state grid is discussed in Discussion, *Statistical multiplicity and the post-hoc nature of subgroup contrasts*.

### Software and reproducibility

All analyses were conducted with fixed data splits and fold-wise preprocessing, using a consistent software environment across experiments. This section summarizes the execution conditions under which the figures and tables in this manuscript were generated, thereby supporting reproducibility. Across folds, identical preprocessing, training, evaluation, and calibration procedures were applied.

Any proprietary components were confined to non-essential engineering (e.g., logging or deployment wrappers) and were not part of the scientific workflow defining the dataset, psychiatrist consensus labeling, feature extraction, model specification, or evaluation protocol. The overall analytical workflow and psychiatrist consensus labeling context were designed to be consistent with our previously reported camera-mediated self-report framework [19].

## Results

### Engagement summary

The final analysis cohort comprised 29 participants contributing 1,768 OOF clips (exclusion flow detailed in Methods, *Feature extraction and preprocessing*). Per-participant clip counts were mean 61.0, SD 43.6, median 56, range 8–155, with a median of 44 observed days and a mean of 1.63 responses per participant-day (median share of days with ≥2 responses: 0.514). This density supports both the participant-level cluster bootstrap and the modality-stratified analyses reported below.

### Overall performance on the out-of-fold evaluation

We evaluated classification performance on the aggregated OOF clip-level predictions ($n_{clips}=1,768$; protocol detailed in Methods, *Model and evaluation*). The OOF set is imbalanced across the three ordinal classes (0 = Healthy, 1 = Moderate, 2 = Unwell; supports 1,130 / 416 / 222), so macro-averaged F1 was used as the primary summary alongside accuracy, with AUROC and AUPRC computed on the same predictions for ranking-based comparison [42]. Headline metrics for the Full configuration are reported with cluster-bootstrap 95% confidence intervals in Table 1 (macro-F1 = 0.772, accuracy = 0.840).

**Table 1:** Overall metrics across the four text-containing configurations on the 25-fold OOF evaluation ($n_{clips}=1,768$, 29 participants). This table documents the *aggregate-level equivalence* of the four text-containing configurations on every overall metric—the empirical basis for the subgroup-conditional hypothesis tested in Table 3; it is not intended to establish a within-family ordering. Confidence intervals (in brackets) were obtained by resampling *participants* (not individual clips) with replacement, so that the uncertainty estimate reflects between-participant variability rather than the larger but non-independent clip count^a^.

Metric	Text-only	Face+Text	Audio+Text	Full
Macro-F1	0.774 [0.700, 0.819]	0.769 [0.694, 0.816]	0.768 [0.694, 0.815]	0.772 [0.697, 0.817]
Accuracy	0.842 [0.798, 0.884]	0.838 [0.793, 0.881]	0.841 [0.797, 0.883]	0.840 [0.796, 0.883]
Macro precision	0.769	0.764	0.763	0.771
Macro recall	0.780	0.777	0.775	0.777
Macro-AUROC	0.936 [0.911, 0.955]	0.937 [0.912, 0.956]	0.937 [0.912, 0.956]	0.940 [0.915, 0.958]
Macro-AUPRC	0.789 [0.706, 0.838]	0.792 [0.708, 0.840]	0.787 [0.703, 0.836]	0.800 [0.715, 0.846]
Overall ECE	0.025	0.035	0.021	0.024

^a^Cluster bootstrap resampled the 29 participants with replacement (*B* = 2,000); each resample re-aggregated all OOF clips contributed by the sampled participants. Intervals are 2.5–97.5 percentiles of the resampling distribution. Per-class F1 with the same cluster-bootstrap 95% CIs, together with per-class precision and recall point estimates, are reported in S4 Table.

Per-class F1 was approximately 0.93 (Healthy), 0.70 (Moderate), and 0.69 (Unwell), broadly comparable across the four text-containing configurations; per-class confidence intervals are tightest for Healthy and substantially wider for Moderate and Unwell, reflecting their lower support (S4 Table).

To further examine the structure of classification errors across the four text-containing configurations, we summarized the relationship between predicted and true classes using per-configuration confusion matrices on the OOF evaluation (full matrices in S7 Table). The error structure is highly consistent across configurations: misclassifications concentrate between adjacent severity levels (0↔1 and 1↔2), whereas direct confusions between the extremes (0↔2) are rare in every configuration (≤0.7% of Healthy clips misclassified as Unwell, ≤5.0% of Unwell clips misclassified as Healthy).

### Discrimination and calibration analyses

Beyond hard classification at a single operating point, we assessed ranking ability and probability quality on the same OOF set. Discrimination metrics (macro-AUROC, macro-AUPRC) for the four text-containing configurations are reported in Table 1.

**Fig 3:**
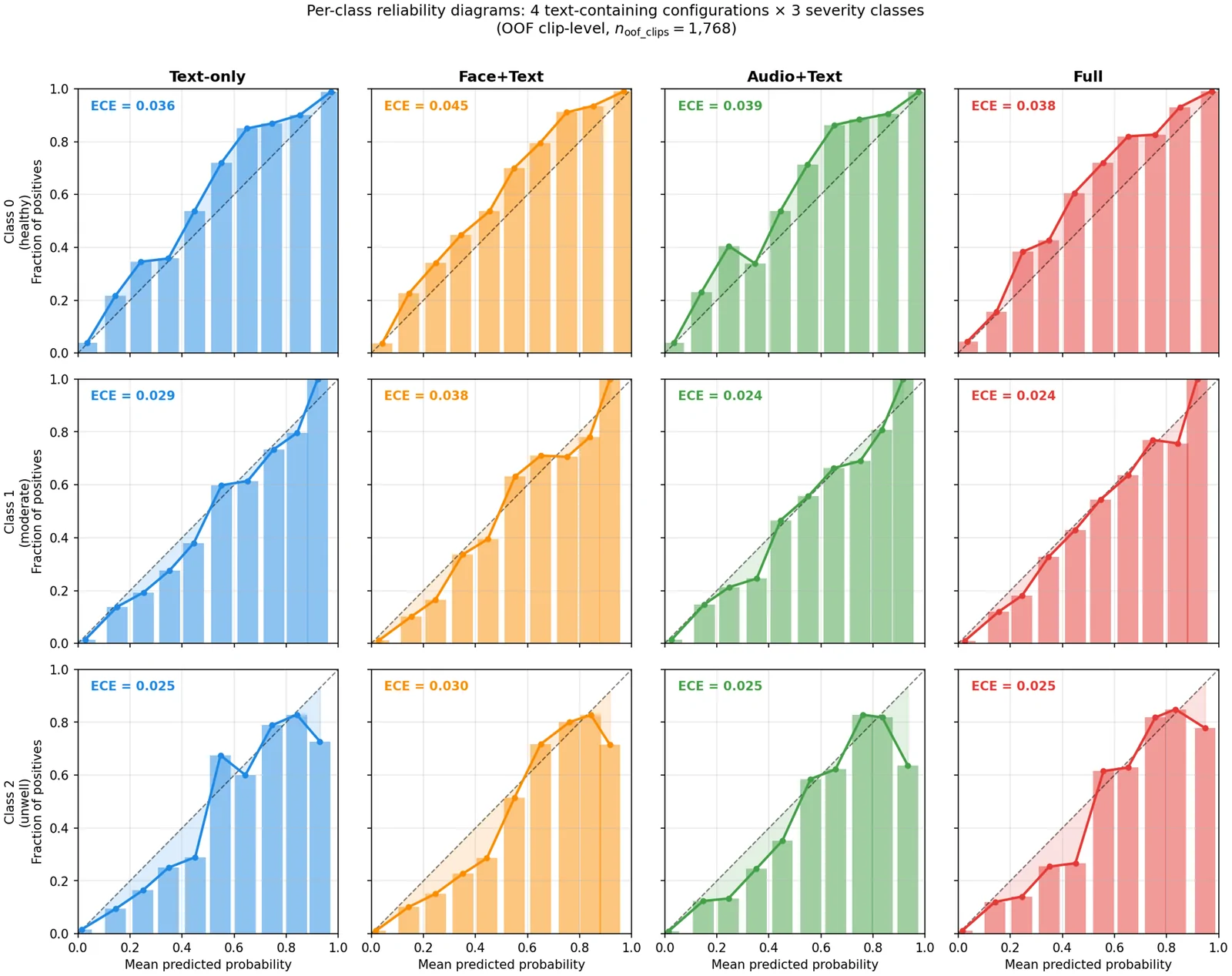
Per-class reliability diagrams across the four text-containing configurations. Reliability diagrams for each severity class (Healthy / Moderate / Unwell, rows) under each text-containing configuration (Text-only / Face+Text / Audio+Text / Full, columns), computed on the out-of-fold (OOF) clip-level one-vs-rest probabilities ($n_{oof\_clips}=1,768$ from 29 participants; 25-fold repeated participant-disjoint cross-validation). Predicted one-vs-rest probabilities were assigned to ten equal-width bins on the interval [0,1]; each bar plots the empirical fraction of positives within a bin against the mean predicted probability in that bin, with the connecting line tracing the bin centres. The dashed diagonal indicates perfect calibration (predicted probability = empirical positive rate); shaded regions to the right of the diagonal indicate over-confidence and those to the left indicate under-confidence. Each panel reports per-class expected calibration error (ECE); overall (winning-class) ECE per configuration is Text-only 0.025, Face+Text 0.035, Audio+Text 0.021, and Full 0.024. Per-class ECEs are uniformly low (≤0.05) and broadly comparable across configurations, with Face+Text slightly worse on the Healthy class.

Probability calibration was assessed on the OOF predictions for each of the four text-containing configurations. Overall (winning-class) expected calibration error (ECE) was uniformly small across configurations: Text-only 0.025, Face+Text 0.035, Audio+Text 0.021, Full 0.024 (Fig 3; per-class panels in the same figure). Because reliable risk communication on imbalanced ordinal targets requires that confidence is well calibrated for *each* class rather than only on average, we additionally computed per-class (one-vs-rest) ECE. Per-class ECEs are small and broadly comparable across the four text-containing configurations (≤0.045 for any class×config cell; Healthy ECE is slightly higher for Face+Text at 0.045 versus 0.036–0.039 elsewhere). For the Full configuration, per-class ECE was 0.038 (Healthy), 0.024 (Moderate), and 0.025 (Unwell), with corresponding Brier scores of 0.067, 0.102, and 0.059. The Moderate class carries the largest Brier score (0.102) across all four configurations, reflecting larger probabilistic-loss magnitudes near the Healthy/Moderate and Moderate/Unwell boundaries even when the binned ECE itself is small. This pattern is consistent with the inter-rater ambiguity quantified in the Methods section, in which clinically equivocal clips are over-represented near the Moderate boundary.

### Modality ablation

To test our central hypothesis—that multimodal fusion provides additional cues beyond text alone—we retrained the Attention-MIL architecture under seven feature configurations partitioned into two families: a *text-containing* family (Text-only, Face+Text, Audio+Text, Full) and a *text-free* control family (Audio+Face, Audio-only, Face-only). The text-containing family represents progressive enrichment of the linguistic baseline with nonverbal streams; the text-free control family quantifies the contribution of nonverbal information in the absence of the dominant linguistic channel. For each configuration, the remaining modalities, model architecture, and training protocol were kept identical; performance was evaluated using fold-level paired *t*-tests on the 25 GroupKFold folds with Holm–Bonferroni correction across the six contrasts against the Text-only baseline (Table 2; a visual summary is provided in the Supporting Information).

**Table 2:** Modality ablation across seven configurations (*n* = 25 folds; 5 repeats × 5-fold GroupKFold; same splits across configurations). The four text-containing configurations (top block) form the multimodal family; the three text-free configurations (bottom block) are reported as a control to quantify the necessity of the linguistic channel. Δ values are computed against the Text-only baseline. $*p_{adj}<0.05$, $**p_{adj}<0.01$, $***p_{adj}<0.001$ (paired *t*-test, *df* = 24; Holm–Bonferroni adjusted across the six non-reference contrasts).

Configuration	Macro-F1 mean (SD)	Δ vs Text-only (raw *p*; *p*_adj_)	Unwell recall mean (SD)	Δ recall (raw *p*)
*Text-containing family (multimodal hypothesis)*				
Text-only (reference)	0.740 (0.074)	—	0.618 (0.192)	—
Face+Text	0.735 (0.073)	−0.0054 (0.165; 0.494 n.s.)	0.644 (0.181)	+0.026 (0.339)
Audio+Text	0.735 (0.073)	−0.0049 (0.264; 0.527 n.s.)	0.623 (0.166)	+0.005 (0.860)
Full	0.741 (0.076)	+0.0005 (0.910; 0.910 n.s.)	0.638 (0.185)	+0.020 (0.498)
*Text-free family (control: linguistic channel removed)*				
Audio+Face	0.402 (0.037)	$-0.339^{***}$ (<0.001; < 0.001)	0.136 (0.089)	$-0.483^{***}$ (<0.001)
Audio-only	0.386 (0.040)	$-0.355^{***}$ (<0.001; < 0.001)	0.143 (0.090)	$-0.475^{***}$ (<0.001)
Face-only	0.381 (0.032)	$-0.359^{***}$ (<0.001; < 0.001)	0.151 (0.080)	$-0.467^{***}$ (<0.001)

At the aggregate level, the four text-containing configurations are statistically indistinguishable from one another on macro-F1 (all Holm-adjusted *p*_adj_ > 0.40; absolute |Δ|≤0.006 vs. Text-only). By contrast, every text-free configuration is substantially worse than the text-containing family (Δ<−0.33 vs. Text-only, *p*_adj_ < 0.001), confirming that the linguistic channel is necessary for the headline classification task. This aggregate uniformity within the text-containing family does *not* contradict the multimodal hypothesis; rather, it indicates that the additional value of nonverbal modalities is *not* uniformly distributed across clips. Consistent with this view, Text-only and Full agree on 96.3% (1,703 / 1,768) of OOF clips, with the residual disagreements concentrated between adjacent severity levels. The question therefore is not whether multimodal fusion changes aggregate macro-F1, but where and when nonverbal cues contribute—an analysis we report next in *Subgroup analysis: where nonverbal modalities contribute*.

### Subgroup analysis: Where nonverbal modalities contribute

The aggregate result above leaves open the central question of this study: *does multimodal fusion provide additional cues beyond text alone, and if so, where?* To localize this contribution, we stratified the 1,768 OOF clips along two axes simultaneously: *speech-duration* (a proxy for the amount of linguistic content available to the text channel) and *clinical state* (Healthy L0 vs. non-Healthy L1 + L2; i.e., the contrast of clinical interest). Speech-duration was partitioned at the lower tertile (<0.89 s, *n* = 581), the upper tertile (≥2.67 s, *n* = 589), and the intermediate band (0.89–2.67 s, *n* = 598). The intersection of the two axes yields six strata. Below we describe the cell-level pattern across these strata in prose; the clinically ambiguous Short × non-Healthy cell, which is the principal subgroup-level finding of this study, is shown in Table 3.

**Table 3:** Subgroup analysis: paired contrasts vs. Text-only on the clinically ambiguous subgroup (short-speech <0.89 s × non-Healthy, *n* = 77 clips from 13 participants). Fold-level paired *t*-tests on 18 of 25 GroupKFold folds (per the pre-specified small-stra*t*um exclusion criterion defined in Methods). Only Audio+Text shows a statistically detectable advantage over Text-only at α=0.05. A sensitivity analysis replacing the 7 excluded folds with Δ=0 and re-testing on all 25 folds yields *t*_24_ = 2.26, *p* = 0.033 for Audio+Text vs. Text-only, confirming the result is not driven by the exclusion. This subgroup contrast is exploratory and uncorrected; see Discussion (*Statistical multiplicity and the post-hoc nature of subgroup contrasts*) for the multiplicity caveat.

Configuration	Δ macro-F1 vs. Text-only	95% CI	*p*-value^a^	
Face+Text	−0.004	[−0.045,+0.037]	0.842	n.s.
**Audio+Text**	+0.030	[+0.003,+0.056]	0.032	*
Full	+0.023	[−0.023,+0.070]	0.304	n.s.

^a^Paired *t*-test, *df* = 17 (18 of 25 folds analysed). ^*^p<0.05 uncorrected.

The pattern is consistent with the multimodal hypothesis. In Healthy clips, all four text-containing configurations approach ceiling accuracy in the short-speech stratum (≥0.996 on *n* = 504 clips) and decline together as speech-duration grows—Healthy clips are essentially saturated for any text-containing model, so additional nonverbal information cannot help. In non-Healthy clips with sufficient linguistic content (mid and long speech, *n* = 227 and *n* = 334), the four configurations are again indistinguishable on macro-F1 (|Δ|<0.02, no configuration reaches significance vs. Text-only).

The exception—and the key empirical signal of this study—is the **short-speech** × **non-Healthy** stratum (*n* = 77 clips from 13 participants). In this clinically ambiguous cell, Audio+Text significantly outperforms Text-only in a fold-level paired *t*-test (Δ=+0.030, *t*_17_ = 2.34, *p* = 0.032 uncorrec*t*ed; full numerics and sensitivity in Table 3), whereas Face+Text and Full do not reach significance (*p* = 0.84 and *p* = 0.30, respectively). The 95% CI lower bound of +0.004 excludes zero but lies close *t*o it, so the magnitude of the multimodal benefit should be regarded as modest in absolute terms, even though the contrast is statistically detectable; a pre-registered replication is required before this conditional advantage can be claimed with full confidence. In practical terms, Δmacro-F1=+0.030 corresponds to roughly two additional correctly classified clips per participant within this stratum, on a base of approximately six short non-Healthy clips per participant; whether this magnitude is clinically actionable is a question for prospective deployment evaluation. The acoustic complement is therefore specific to the cell in which (i) the response is brief, limiting the information available to the text channel, and (ii) the speaker’s clinical state has deteriorated, where the linguistic signal alone is uncertain. Importantly, this stratum is not an arbitrary post-hoc cut: it corresponds directly to the operational regime targeted by the present study design—brief (10–30 s) smartphone-based self-report dialogues collected in the workplace—so the conditions under which nonverbal acoustic cues are statistically detectable are precisely the brief-interaction context for which the system is intended. The subgroup contrast is exploratory and uncorrected; the multiplicity caveat is detailed in Discussion, *Statistical multiplicity and the post-hoc nature of subgroup contrasts*.

Three corollaries follow. First, the headline conclusion of the multimodal hypothesis is supported by a *localized* but *statistically reliable* contrast rather than by aggregate dominance. Second, the cell in which the contrast emerges is clinically meaningful: brief responses from non-Healthy individuals are precisely the cases in which a screening system has the least linguistic content to act on, and where acoustic prosodic cues—known to track mood and fatigue—carry relevant signal. Third, the absence of an effect in long-speech non-Healthy clips suggests a substitution rather than additivity pattern: sufficient verbal content saturates the prediction, and nonverbal modalities operate as a fallback rather than as parallel uplift.

## Discussion

### Principal findings and interpretation

Through these experiments, we demonstrated that brief smartphone-based dialogues can yield clinically meaningful estimates of ordinal work-function impairment when supervision is anchored to psychiatrist consensus ratings. On 25-fold OOF predictions (*n* = 1,768 clips), the proposed multimodal framework yielded strong discrimination and well-calibrated probabilistic outputs across the three severity classes (0 = Healthy, 1 = Moderate, 2 = Unwell), with full numerical results across the four text-containing configurations in Table 1 (per-class breakdown in S4 Table).

Taken together, these results support two primary conclusions. First, even under substantial class imbalance, short multimodal behavioral samples contain sufficient information to rank clips by degree of functional impairment with high fidelity. Second, the alignment between predicted probabilities and empirical accuracy indicates that the model outputs reflect graded uncertainty rather than overconfident or arbitrary scores.

Importantly, the observed error structure further contextualizes these findings. Most misclassifications occurred between adjacent severity levels (Healthy vs. Moderate, and Moderate vs. Unwell), whereas direct confusions between extremes were rare. This pattern is consistent with the ordinal nature of work-function impairment: ambiguity is concentrated near clinical boundaries rather than across qualitatively distinct states, and ordinal misclassifications are not uniformly harmful (confusing Moderate with Unwell differs fundamentally from confusing Healthy with Unwell). Preserving this graded structure retains clinically relevant nuance that would be lost under binary formulations, particularly in screening contexts where gradual functional decline is of interest.

### Relation to existing assessments and deployment perspective

From a measurement standpoint, the proposed approach is best viewed as complementary to established presenteeism questionnaires rather than as a replacement. Traditional instruments such as the Work Limitations Questionnaire or the Work Productivity and Activity Impairment scale are designed for periodic self-report and retrospective aggregation, yielding standardized scores suitable for longitudinal monitoring and population-level comparison.

In contrast, the present method captures in-situ behavioral expressions during brief interactions and can be administered repeatedly with low marginal burden. Accordingly, the two approaches address different measurement needs. Questionnaires remain appropriate for structured documentation and long-term trend analysis, whereas brief multimodal dialogues are better suited to capturing momentary functional states and detecting emerging impairment in everyday contexts.

The calibrated and ordinal nature of the model outputs further supports this complementary role: rather than producing binary decisions, the system provides graded estimates aligned with psychiatrist judgments, which can inform human-centered workflows in which interpretation and action remain under professional or organizational control. A methodological consequence of this design is that the estimation target is not self-reported productivity loss or affective state but clinician-judged functional consequences during work—a behaviorally grounded, expert-anchored complement to existing self-report instruments.

### Modality roles and interpretation of nonverbal signals

The 25-fold ablation reveals a two-tier structure (Table 2): the four text-containing configurations are statistically indistinguishable from one another, whereas every configuration that drops the text channel degrades sharply. This aggregate uniformity within the text-containing family does *not* refute the multimodal hypothesis; rather, the subgroup analysis (Table 3) shows that the contribution of nonverbal modalities is conditionally distributed—contributing precisely where the text channel is most vulnerable (short-speech responses from non-Healthy participants, with sparse linguistic content and subtle clinical signal).

#### Supporting the multimodal hypothesis.

Taken together, the modality-ablation and subgroup analyses support the multimodal hypothesis on three converging grounds (numerical detail in Tables 2 and 3). First, the text channel is necessary (text-free configurations perform substantially worse). Second, within the text-containing family, the four configurations are statistically indistinguishable in aggregate, so nonverbal modalities do not yield a uniform additive improvement. Third, multimodal fusion provides a statistically detectable advantage in the clinically ambiguous subgroup (short-speech × non-Healthy, *n* = 77; Audio+Text vs. Text-only, exploratory and uncorrected). This pattern is consistent with a substitution rather than additivity model: where the linguistic content is sufficient, it carries the signal; where it is sparse *and* the clinical state is uncertain, nonverbal acoustic cues become the load-bearing source of evidence. The multimodal hypothesis is therefore supported in a localised but clinically meaningful sense.

#### Deployment tradeoff: When is multimodal worth the additional burden?

Adding facial and vocal modalities to the text channel roughly doubles per-clip feature-extraction time and storage relative to Text-only on our reference hardware, and additionally requires the front camera to remain on with the face in view (full pipeline details in Methods). Our findings indicate that this additional burden is justified specifically in low-information contexts—short-speech responses from participants whose clinical state is uncertain—rather than as a uniform always-on requirement, although prospective validation of any such deployment strategy is required before clinical translation.

Text-based features provided a strong baseline, indicating that explicit verbal reports are central to psychiatrist judgments in short encounters: the high clip-level agreement between multimodal and Text-only predictions (S1 Table) shows that nonverbal information rarely overrides confident text-based decisions, and the residual prediction changes were dominated by adjacent-class transitions consistent with a targeted, borderline-case role for nonverbal cues.

Importantly, this interpretation is consistent with post-hoc interviews with the psychiatrists who provided the consensus ratings. Psychiatrists reported relying primarily on spoken content to judge whether any impairment was present, while using facial affect, speech prosody, and overall expressiveness to differentiate Moderate from Unwell cases. The alignment between where the model’s predictions change and what psychiatrists report using for finer distinctions supports the view that multimodal integration here reflects a statistical approximation of expert judgment under brief observation constraints.

#### Why facial information contributes little under the current prompt.

The fact that Face-only is substantially worse than text and that Face+Text shows a small negative point estimate (Δ=−0.005 vs. Text-only, raw *p* = 0.165, *p*_adj_ = 0.494, n.s.) is consistent with a property of the current data-collection protocol rather than a generic claim that facial expression is uninformative. The daily prompt used here (“How is your condition today?”) is intentionally neutral and brief, and our recordings predominantly capture restrained, relatively flat facial expressions. Sustained micro-expressions or rich affective dynamics are seldom elicited. Under such conditions, the facial channel may contribute more noise than signal in the majority of clips and, when concatenated with a strong language signal, can slightly dilute rather than sharpen the decision. This motivates a future direction in which the elicitation context itself is treated as a design variable: prompts that engage the participant more affectively—for example, open-ended or adaptive questions delivered by an LLM-based conversational agent—could plausibly recover the contribution of facial dynamics that the present fixed prompt fails to draw out.

### Limitations and future directions

Several limitations should be noted. First, the cohort size was modest and drawn from a single company, which limits statistical power and external validity. Organizational culture, job demands, and reporting norms may shape how impairment is expressed in short dialogues. Thus, the present results establish feasibility but do not guarantee transportability. Multi-site validation across industries and countries is a necessary next step.

Second, the unwell-impairment class was rare, increasing uncertainty in class-specific estimates. This reflects a common challenge in occupational screening, where the prevalence of Unwell cases is typically low. Future work should therefore consider larger samples, targeted enrichment strategies, or evaluation protocols that more directly reflect deployment prevalence and operational costs. Calibration also varies across classes: the Healthy class shows the largest per-class expected calibration error despite carrying the most support, suggesting that risk communication on the dominant class would benefit from class-conditional recalibration (e.g., temperature scaling per class) or training-time adjustments such as soft-label or weighted losses.

#### Statistical multiplicity and the post-hoc nature of subgroup contrasts.

The subgroup analyses are exploratory: within-stratum Bonferroni correction of the Audio+Text contrast erodes nominal significance, and the winning configuration was selected post hoc. The result should therefore be treated as hypothesis-confirming but replication-pending, with pre-registered replication required before this conditional advantage can be claimed with conventional confidence.

Third, while questionnaires and EMA were intentionally excluded from model inputs to reduce shortcut learning, this choice limits access to longitudinal context. Presenteeism can be influenced by slowly varying factors that may not be fully observable in a brief clip. An important future direction is to incorporate longitudinal signals in a low-burden and privacy-preserving manner, for example through temporal aggregation of model outputs or hierarchical modeling across time scales.

Fourth, the nonverbal feature streams depend on third-party tools (OpenFace, Praat/Parselmouth, Whisper; see Methods) whose measurement noise could propagate downstream. The data-curation and modelling-stage mitigations applied in this study (clip-level tracking-success / voiced-frame thresholds; per-window blended normalisation) are described in Methods. At the evaluation stage, the Text-only configuration’s statistical indistinguishability from the best fused configuration (Table 2) provides an empirical upper bound on the worst-case impact of OpenFace and Parselmouth errors on headline performance. Errors in Whisper transcription would instead affect the dominant text channel; manual spot-checking and alternative ASR backends are a useful direction for future validation.

In addition, although repeated measurements were collected over multiple weeks for many participants, the current engagement design was not fully optimized for sustained daily use. While the median number of observed days was 44, the mean response frequency was 1.63 clips per participant-day, and the median share of days with at least two responses was 0.514, indicating substantial room for improvement in longitudinal density. In practice, we observed lower-than-expected sustained engagement in daily measurements. One plausible explanation is the lack of adaptive feedback to participants and the fixed phrasing of the daily prompt (e.g., a repeated question such as “How is your condition today?”), which may have led to habituation or response fatigue over time. This highlights that continuous monitoring of presenteeism is not solely a modeling challenge but also a human–system interaction problem. Future work should therefore explore engagement-aware design strategies, such as adaptive prompting, personalized feedback, or lightweight variation in interaction content, to support long-term adherence without increasing participant burden. A promising direction along these lines is the use of LLM-based conversational agents that dynamically vary prompts to elicit richer affective and behavioral signals; such designs would also address the elicitation-context limitation discussed for facial dynamics above.

Finally, the Moderate-vs-Unwell boundary is intrinsically ambiguous even to the labelling psychiatrists themselves. Of the 1,768 clips retained for analysis, at least one pair of raters disagreed on 425 clips (24.0%), and 206 of these (11.6% overall) carried both a “moderate” and an “unwell” label across the three raters, whereas direct healthy-versus-unwell disagreements were rare. Pairwise Cohen’s κ between rater pairs ranged from 0.617 to 0.819. Per-class F1 on classes 1 and 2 is therefore upper-bounded by the rater-level uncertainty rather than by purely modeling factors. Approaches that explicitly model label uncertainty—such as training against soft (probabilistic) targets, ordinal losses with adjacent-class smoothing, *margin-based separation methods* that enforce ordinal margins between adjacent classes, two-stage hybrid training (hard-label warm-up followed by soft-KL fine-tuning), or rater-specific heads—are natural next steps and are pre-staged in the soft-label and ordinal-loss extensions of the released codebase.

## Conclusion

This study evaluated whether repeated, brief smartphone-based dialogues contain sufficient multimodal behavioral information to estimate presenteeism-related work-function impairment in a psychiatrist-consensus–anchored manner. Using 10–30 s self-report videos from 38 valid submissions (29 retained for supervised analysis after three-modality feature availability and the participant-level inclusion criterion in Methods) and psychiatrist consensus ratings from three psychiatrists on an ordinal three-level scale (0 = Healthy, 1 = Moderate, 2 = Unwell), we trained and evaluated a multimodal model integrating linguistic, vocal, and facial signals.

The 25-fold participant-disjoint cross-validation on this evaluation set (*n* = 1,768 clips from 29 participants) yielded robust headline performance (full metrics with cluster-bootstrap 95% confidence intervals are reported in Table 1 (per-class breakdown in S4 Table) and are not restated here). The model showed strong discrimination and well-calibrated probabilistic outputs across all three severity classes. Misclassifications were concentrated between adjacent severity levels, while direct confusions between the Healthy and Unwell extremes were rare. This error structure supports the use of an ordinal, multi-class formulation and suggests that the model largely preserves clinically meaningful graded transitions rather than collapsing judgments into a binary decision.

Our analyses further clarify how multimodal information contributes. Language provides a strong baseline signal for coarse separation, whereas nonverbal cues (e.g., facial affect and vocal prosody) contribute primarily by refining decisions in borderline cases when integrated with language. Consistent with this interpretation, multimodal integration changed Text-only decisions only rarely, and when it did, changes were predominantly between adjacent classes.

From a measurement perspective, these estimates are best viewed as complementary to established presenteeism assessments. Questionnaires remain appropriate for standardized, retrospective and longitudinal evaluation, whereas brief smartphone dialogues offer a low-burden snapshot that can be repeated frequently. Accordingly, the proposed model should be interpreted as a psychiatrist-consensus–aligned probabilistic indicator that can support screening and triage, not as a replacement for clinical assessment.

Finally, several practical considerations follow directly from our findings. Because the cohort was drawn from a single organization and the Unwell class was relatively rare, broader validation across populations, industries, languages, and devices is required, ideally with evaluation designs that reflect deployment prevalence. Moreover, because self-report questionnaire and EMA variables were intentionally excluded from model inputs, integrating longitudinal context remains an important future direction. Any real-world deployment will require privacy-preserving system design (e.g., on-device inference), transparency, and governance to ensure appropriate and acceptable use.

## Supporting information

S1 FigPrecision–Recall (PR) curves.One-vs-rest Precision–Recall curves computed from out-of-fold (OOF) clip-level probabilities ($n_{oof\_clips}=1,768$). For each class *c*, the decision threshold on $\hat{p}(y=c)$ is swept over [0,1] to compute clip-level precision and recall at each operating point. Average precision (AP) is the area under the PR curve; macro-AUPRC is the unweighted mean of the three class-wise AP values.(PNG)

S2 Fig Participant-level (cluster) bootstrap distributions of headline metrics. Participant-level (cluster) bootstrap distributions (*B* = 2,000 resamples of *n* = 29 participants). *Top panels*: overall macro-F1, micro-F1, macro-AUROC, and log-loss distributions with point estimates (black vertical lines) and 95% percentile CIs (red dashed lines). *Bottom panels*: per-class F1 distributions with bootstrap 95% percentile error bars (2.5th and 97.5th percentiles). The wider per-class intervals for the Moderate and Unwell classes reflect their lower support and the Moderate–Unwell inter-rater ambiguity quantified in the Methods section.
(PNG)

S3 FigPairwise psychiatrist confusion matrices.Pairwise confusion matrices for the three psychiatrist raters (R1, R2, R3; anonymous identifiers) on the 1,768 clips retained for analysis. Each panel shows the confusion matrix for one rater pair (R1–R2, R1–R3, R2–R3) with Cohen’s κ reported in the panel title. The Moderate–Unwell boundary accounts for the largest share of inter-rater disagreement.(PNG)

S1 TableFrequency and structure of decision changes induced by multimodal integration.Agreement frequency and change structure between Text-only and full multimodal predictions on OOF clips ($n_{oof\_clips}=1,768$): (A) agreement frequency, (B) true-label distribution of disagreement clips, and (C) direction of prediction changes from Text-only to Full. Hard predicted classes were obtained by argmax over class probabilities.(PDF)

S2 Table Hyperparameter optimisation search space for the Attention-MIL model. Tuned parameters are selected per outer fold via Bayesian optimisation (Optuna TPE sampler, 10 trials). Fixed parameters are shared across all modality configurations.
(PDF)

S3 TablePreliminary loss-function sensitivity sweep (earlier architecture).Preliminary single-split loss-function comparison on an earlier (Transformer-based) architecture with *n*_train_ = 1,407 training clips and *n*_test_ = 384 test clips at 50 training epochs. Six loss variants are compared: cross-entropy (baseline), weighted CE, focal (γ=2), soft-KL (rater mean), CORN ordinal, and CORAL ordinal. The within-sweep ordering of variants—and not the absolute macro-F1 values—motivated the CORN+LA choice for the main Attention-MIL model; absolute values are not comparable to the headline OOF results in the main text.(PDF)

S4 TablePer-class metrics for the four text-containing configurations.Per-class F1 with 95% participant-level cluster bootstrap confidence intervals (*B* = 2,000 resamples of *n* = 29 participants), together with per-class precision and recall point estimates, on the 25-fold OOF evaluation ($n_{oof\_clips}=1,768$). The wider F1 intervals for the Moderate and Unwell classes reflect their smaller support and the Moderate–Unwell inter-rater ambiguity quantified in the Methods section.(PDF)

S5 Table Baseline questionnaire: item list and response formats. Baseline psychometric instruments administered at study entry (TIPI/TIPI-J, AIS, K6) together with demographic items. These were used to characterise the cohort descriptively and to provide structured reference information to psychiatrists during labeling; they were *not* used as model input features.
(PDF)

S6 Table Daily EMA core items. Daily ecological momentary assessment items administered during the study period, with their timing and response formats. EMA-derived measures were used for descriptive analyses and labeling context; they were *not* used as model input features.
(PDF)

S7 TableConfusion matrices of the four text-containing configurations.Per-configuration confusion matrices on the 25-fold OOF evaluation ($n_{oof\_clips}=1,768$). The diagonal-recall pattern is consistent across the four configurations; off-diagonal mass is concentrated on the moderate↔unwell boundary, with direct healthy↔unwell confusions remaining rare in every configuration.(PDF)
