# Supplementary material for: Estimating presenteeism from repeated smartphone-based multimodal behavioral responses
Source: PLoS One. 2026 Sep 16;21(9):e0354342. doi: 10.1371/journal.pone.0354342 (PMC13581304; doi:10.1371/journal.pone.0354342)
Supplement: Supplementary file 1 [file pone.0354342.s004.pdf]

**(A) Agreement frequency**	
Predictions agree	1,703 (96.3%)
Predictions disagree	65 (3.7%)
**(B) True labels among disagreement cases ($n_{\text{disagreement\_clips}} = 65$)**	
Healthy (0)	23
Moderate (1)	25
Unwell (2)	17
**(C) Direction of prediction changes (Text-only → Full)**	
Healthy → Moderate	20
Healthy → Unwell	3
Moderate → Healthy	16
Moderate → Unwell	7
Unwell → Healthy	2
Unwell → Moderate	17

Hard predicted classes were obtained by argmax over class probabilities.
