# Supplementary material for: Estimating presenteeism from repeated smartphone-based multimodal behavioral responses
Source: PLoS One. 2026 Sep 16;21(9):e0354342. doi: 10.1371/journal.pone.0354342 (PMC13581304; doi:10.1371/journal.pone.0354342)
Supplement: Supplementary file 2 [file pone.0354342.s005.pdf]

| Parameter | Type / Range | Notes |
|---|---|---|
| **Tuned by Optuna (per outer fold)** | | |
| Hidden dimension (`hidden`) | Categorical: {64, 128, 256} | Attention-MIL stream width |
| Dropout rate (`dropout`) | Uniform: [0.10, 0.40] | Applied in attention + head |
| Learning rate (`lr`) | Log-uniform: $[10^{-4}, 3 \times 10^{-3}]$ | AdamW |
| Batch size (`bs`) | Categorical: {8, 16, 24} | Clip-level mini-batch |
| Training epochs (`epochs`) | Integer: [10, 30] | Outer-fold retraining |
| **Fixed across all configurations** | | |
| Weight decay | $10^{-4}$ | AdamW |
| Logit-adjustment $\tau$ | 1.0 | Class-prior correction (LA) |
| Modality dropout $p$ | 0.3 | Face & voice streams |
| CORAL aux-loss weight | 0.2 | Text stream only |
| Optimisation trials (`N_OUTER_TRIALS`) | 10 | Bayesian (TPE sampler) |
| Inner CV splits | 3-fold GroupKFold | Participant-disjoint |
