# Supplementary material for: Estimating presenteeism from repeated smartphone-based multimodal behavioral responses
Source: PLoS One. 2026 Sep 16;21(9):e0354342. doi: 10.1371/journal.pone.0354342 (PMC13581304; doi:10.1371/journal.pone.0354342)
Supplement: Supplementary file 3 [file pone.0354342.s006.pdf]

Variant	macro-F1	F1 (Heal.)	F1 (Mod.)	F1 (Unwell)	ord. MAE	ECE (Mod.)	ECE (mean)
Cross-entropy (baseline)	0.607	0.855	0.612	0.353	0.281	0.231	0.162
Weighted CE	0.618	0.866	0.575	0.414	0.289	0.232	0.164
Focal ($\gamma = 2$)	0.618	0.853	0.573	**0.429**	0.294	**0.131**	**0.096**
Soft-KL (rater mean)	0.481	0.777	0.445	0.219	0.430	0.181	0.159
CORN (ordinal)	0.609	0.840	0.631	0.357	0.318	0.223	0.171
**CORAL (ordinal)**	**0.629**	**0.872**	**0.661**	0.355	**0.255**	0.191	0.141

Bold values mark the best variant within each column. ECE values are per-class (one-vs-rest) expected calibration errors.
