# Supplementary material for: Estimating presenteeism from repeated smartphone-based multimodal behavioral responses
Source: PLoS One. 2026 Sep 16;21(9):e0354342. doi: 10.1371/journal.pone.0354342 (PMC13581304; doi:10.1371/journal.pone.0354342)
Supplement: Supplementary file 4 [file pone.0354342.s007.pdf]

Class	Support	Text-only	Face+Text	Audio+Text	Full
Block A: Per-class F1 with 95% cluster bootstrap CI					
0 (healthy)	1,130	0.927 [0.889, 0.955]	0.926 [0.885, 0.955]	0.931 [0.894, 0.958]	0.926 [0.886, 0.956]
1 (moderate)	416	0.703 [0.619, 0.782]	0.701 [0.619, 0.778]	0.702 [0.613, 0.783]	0.704 [0.619, 0.782]
2 (unwell)	222	0.691 [0.473, 0.800]	0.680 [0.480, 0.795]	0.671 [0.467, 0.785]	0.687 [0.474, 0.802]
Block B: Per-class precision / recall (point estimates)					
0 (healthy) precision	—	0.949	0.952	0.953	0.951
0 (healthy) recall	—	0.906	0.901	0.910	0.904
1 (moderate) precision	—	0.662	0.654	0.664	0.654
1 (moderate) recall	—	0.750	0.755	0.745	0.762
2 (unwell) precision	—	0.697	0.685	0.671	0.708
2 (unwell) recall	—	0.685	0.676	0.671	0.667
