# Supplementary material for: Estimating presenteeism from repeated smartphone-based multimodal behavioral responses
Source: PLoS One. 2026 Sep 16;21(9):e0354342. doi: 10.1371/journal.pone.0354342 (PMC13581304; doi:10.1371/journal.pone.0354342)
Supplement: Supplementary file 5 [file pone.0354342.s008.pdf]

Domain	Item (English translation)	Response format
Demographics	Age; Gender; Education; Job role; Working hours; Commuting time; Sleep duration; Annual income	Numeric or categorical
Personality (Big Five, 10 items; TIPI/TIPI-J)	"I see myself as …(1) extraverted, enthusiastic; (2) critical, quarrelsome; (3) dependable, self-disciplined; (4) anxious, easily upset; (5) open to new experiences; (6) reserved, quiet; (7) sympathetic, warm; (8) careless, disorganized; (9) calm, emotionally stable; (10) conventional, uncreative."	7-point Likert scale (1=Strongly disagree–7=Strongly agree)
Sleep (Athens Insomnia Scale; AIS)	Sleep induction; Awakenings during the night; Final awakening; Total sleep duration; Overall sleep quality; Daytime well-being; Daytime functioning; Daytime sleepiness	4-point scale (0–3 per item)
Psychological distress (K6)	"In the past 30 days, how often did you feel nervous, hopeless, restless or fidgety, so depressed that nothing could cheer you up, that everything was an effort, or worthless?"	5-point scale (0=None–4=Always)

TIPI/TIPI-J (Gosling et al., 2003; Oshio et al., 2012); AIS validation (Soldatos et al., 2000; Okajima et al., 2013); K6 validation (Kessler et al., 2002).
