# Supplementary material for: Estimating presenteeism from repeated smartphone-based multimodal behavioral responses
Source: PLoS One. 2026 Sep 16;21(9):e0354342. doi: 10.1371/journal.pone.0354342 (PMC13581304; doi:10.1371/journal.pone.0354342)
Supplement: Supplementary file 6 [file pone.0354342.s009.pdf]

Item	Description	Timing / Format
Location	Home / Office / Outside (incl. commuting)	Pre-/Ad hoc-/End; single choice
Work status	Pre-work / At work / Post-work / Holiday	Same as above
Previous night's sleep	Hours slept last night	Numeric (hours)
Affect (Russell's model)	Valence and arousal plotted on 2D plane	Continuous (2D point)[a]
Video prompt	"How are you feeling today?" (face+speech)	10–30 s video
Workload factor	Choose more relevant workload type (A vs. B)	End of day; single choice
Job performance	"Rate today's job performance (baseline=100%)."	Numeric (1–100%)
Working hours	Total working hours today	Numeric (hours)
Emotional events	Describe any remarkable events	Free text (optional)

[a] Russell's circumplex model of affect (Russell, 1980; Posner et al., 2005).
