# Supplementary material for: Estimating presenteeism from repeated smartphone-based multimodal behavioral responses
Source: PLoS One. 2026 Sep 16;21(9):e0354342. doi: 10.1371/journal.pone.0354342 (PMC13581304; doi:10.1371/journal.pone.0354342)
Supplement: Supplementary file 7 [file pone.0354342.s010.pdf]

**Config**	**True \ Predicted**	**Healthy (0)**	**Moderate (1)**	**Unwell (2)**
Text-only	Healthy (n=1,130)	1024 (90.6%)	100 (8.8%)	6 (0.5%)
	Moderate (n=416)	44 (10.6%)	312 (75.0%)	60 (14.4%)
	Unwell (n=222)	11 (5.0%)	59 (26.6%)	152 (68.5%)
Face+Text	Healthy (n=1,130)	1018 (90.1%)	105 (9.3%)	7 (0.6%)
	Moderate (n=416)	40 (9.6%)	314 (75.5%)	62 (14.9%)
	Unwell (n=222)	11 (5.0%)	61 (27.5%)	150 (67.6%)
Audio+Text	Healthy (n=1,130)	1028 (91.0%)	94 (8.3%)	8 (0.7%)
	Moderate (n=416)	41 (9.9%)	310 (74.5%)	65 (15.6%)
	Unwell (n=222)	10 (4.5%)	63 (28.4%)	149 (67.1%)
Full	Healthy (n=1,130)	1021 (90.4%)	105 (9.3%)	4 (0.4%)
	Moderate (n=416)	42 (10.1%)	317 (76.2%)	57 (13.7%)
	Unwell (n=222)	11 (5.0%)	63 (28.4%)	148 (66.7%)
