# Supplementary material for: Estimating presenteeism from repeated smartphone-based multimodal behavioral responses
Source: PLoS One. 2026 Sep 16;21(9):e0354342. doi: 10.1371/journal.pone.0354342 (PMC13581304; doi:10.1371/journal.pone.0354342)
Supplement: Supplementary file 8 [file pone.0354342.s012.pdf]

Response to Reviewers

PONE-D-26-07436

Estimating presenteeism from repeated smartphone-based multimodal behavioral responses

2026-05-15

We thank the academic editor and both reviewers for their thoughtful, constructive critiques. Every concern raised has been addressed in the revised manuscript, and in several cases the revisions led us to substantively re-examine our central claim about multimodal fusion. We summarise the major changes below, and then respond to each comment in turn.

Summary of major changes since the original submission. The evaluation has been re-anchored to a canonical, participant-disjoint out-of-fold (OOF) set of $n = 1{,}768$ clips from 29 participants under a 25-fold repeated GroupKFold protocol (5 repeats $\times$ 5 folds); all reported numbers come from this single, fixed evaluation set. The classifier was rebuilt as a three-stream Attention-MIL model with a CORN ordinal head and logit adjustment for class imbalance, and statistical rigour has been deepened so that every headline metric carries a participant-level cluster-bootstrap 95% CI ($B = 2{,}000$ resamples of the 29 participants), with the modality-ablation family of six paired contrasts against the Text-only baseline reported under Holm–Bonferroni correction (Methods, *Model and evaluation*; Table 1 and S4 Table; Table 2). **A key aggregate finding is that the four text-containing configurations (Full / Audio+Text / Face+Text / Text-only) are statistically indistinguishable from one another on macro-F1** ($|\Delta| \leq 0.006$ vs. Text-only, all Holm-adjusted $p_{\text{adj}} > 0.40$), while every text-free configuration performs substantially worse ($\Delta < -0.33$, $p_{\text{adj}} < 0.001$). We therefore revise our original interpretation of a broad aggregate multimodal advantage and reframe the study around an explicit *multimodal hypothesis* ("nonverbal modalities provide additional cues beyond text alone, particularly where linguistic cues are sparse"), partially supported in a subgroup analysis in a clinically ambiguous subgroup—short-speech $\times$ non-Healthy ($n = 77$)—where Audio+Text outperforms Text-only on macro-F1 ($\Delta = +0.030$, $t_{17} = 2.34$, $p = 0.032$ uncorrected; Results, *Subgroup analysis: where nonverbal modalities contribute*; multiplicity caveat in *Statistical multiplicity and the post-hoc nature of subgroup contrasts*). Per-class calibration (ECE + Brier) is now reported alongside the overall ECE, and the Supporting Information has been restructured into 3 figures (S1–S3 Fig) and 7 tables (S1–S7 Table) per the PLOS ONE policy on separate SI files (R0-7); the editorial/administrative requirements (R0-1 through R0-8) are addressed in detail below and in the cover letter.

Editor's Journal Requirements (R0-1 through R0-8)

Reviewer comment: *Please ensure that your manuscript meets PLOS ONE's style requirements, including those for file naming.*

Response. We have followed the PLOS ONE LaTeX template throughout. File naming has been aligned with the conventions in the PLOS ONE submission guide. The Supporting Information has been extracted into a separate file (`supporting_information.tex`; rendered as `Supporting_Information.pdf`) and is uploaded with file type *Supporting Information*.

Reviewer comment: *Please note that PLOS ONE has specific guidelines on code sharing for submissions in which author-generated code underpins the findings.*

Response. Author-generated code underlying all reported analyses—the Attention-MIL training pipeline, the 25-fold repeated GroupKFold evaluation, the cluster-bootstrap routines, and the modality-ablation Holm–Bonferroni framework—is released on the project's public GitHub repository under a permissive open-source licence, and is sufficient to reproduce all numerical figures and tables in the manuscript subject to the data-access conditions in R0-4.

Reviewer comment: *Please provide an amended funding statement that declares all funding or sources of support during this study, and include the statement "There was no additional external funding received for this study."*

Response. The amended Funding Statement is provided in the cover letter and re-stated here for completeness:

> SD received a collaborative research grant from Sojitz Corporation through a collaborative research agreement. SD also received funding from the Japan Agency for Medical Research and Development (AMED; grant number JP25he2822009). SD further received support from JSPS KAKENHI (grant number 24K13524). Sojitz Corporation, DG COMMUNICATIONS Co., Ltd., and Minds in Co., Ltd. provided support in the form of salaries for authors [SD, MH, KH, YI, NK, SM], but did not have any additional role in the study design, data collection and analysis, decision to publish, or preparation of the manuscript. The specific roles of these authors are articulated in the 'author contributions' section. **There was no additional external funding received for this study.**

Reviewer comment: *Please provide non-author contact information (phone/email/hyperlink) for a data access committee, ethics committee, or other institutional body to which data requests may be sent.*

Response. We have nominated the Medical Area Support Room (Research Support), Institute of Medicine, University of Tsukuba, as the non-author institutional point of contact for data access. The full contact details are reproduced in the cover letter:

> Medical Area Support Room (Research Support), Institute of Medicine, University of Tsukuba.
> Email: `sien.ningenss@un.tsukuba.ac.jp`
> Tel: +81-29-853-3022 Fax: +81-29-853-6309
> Address: 1-1-1 Tennodai, Tsukuba, Ibaraki 305-8575, Japan

The Support Room is an institutional body independent of the authors and will respond to legitimate research data requests subject to the data-sharing agreements consistent with the participants' informed consent. Aggregate analysis-level data (out-of-fold predictions, fold-level metric vectors, and cluster-bootstrap distributions) sufficient to reproduce the headline figures and tables are made available via the same contact.

Reviewer comment: *Please be informed that funding information should not appear in the Acknowledgments section or other areas of your manuscript.*

Response. All funding-related text has been removed from the Acknowledgments and from the body of the manuscript. The original funding-statement wording in the Acknowledgments is retained only in tracked-change comments for the editor's reference and does not appear in the rendered manuscript text.

Reviewer comment: *Please provide an amended Funding Statement declaring this commercial affiliation, as well as an updated Competing Interests Statement declaring the affiliations and adherence to PLOS ONE policies on sharing data and materials.*

Response. The updated Competing Interests Statement is provided in the cover letter and reproduced here:

> Authors KH, YI, and NK are employed by Sojitz Corporation; SM is employed by DG COMMUNICATIONS Co., Ltd.; SD and MH are affiliated with Minds in Co., Ltd. (in addition to their University of Tsukuba positions). Part of this study uses technology related to a patent (Publication No. JP-A-2026-45820; PCT/JP2025/013259). The patent applicants are Sojitz Corporation, the University of Tsukuba, and DG COMMUNICATIONS Co., Ltd. Among the authors, SD, MH, TN, DH, KH, YI, NK, and KS are listed as inventors. **This does not alter our adherence to PLOS ONE policies on sharing data and materials.**

Reviewer comment: *We notice that your supplementary tables are included in the manuscript file. Please remove them and upload them with the file type "Supporting Information". Please ensure that each Supporting Information file has a legend listed in the manuscript after the references list.*

Response. The Supporting Information has been removed from the manuscript body and is submitted as a separate file (`supporting_information.tex`; rendered as `Supporting_Information.pdf`) with file type *Supporting Information*. The SI now contains three figures (S1–S3 Fig) and seven tables (S1–S7 Table). Their legends are listed in the manuscript file immediately after the references list, as required by the PLOS ONE submission policy.

Reviewer comment: *If the reviewer comments include a recommendation to cite specific previously published works, please review and evaluate these publications.*

Response. The reviewers did not recommend additional citations to specific previously published works; no new citations have been added on this basis. All citations added in this revision arise from the methodological and analytic changes described in the responses to R1-1 through R2-8 (e.g., Akiba 2019 for Optuna, Holm 1979 for the multiple-comparison correction, Cao 2020 for CORAL, Shi 2023 for CORN, Menon 2021 for logit adjustment, Ilse 2018 for Attention-MIL).

Reviewer #1

Reviewer comment: *A weak point in the work is the low results, as shown in S4 Table for classes 1 and 2. A psychiatrist's ratings comparison could explain this result. Are the psychiatrists' ratings also mixing classes 1 and 2? How can this be overcome?*

Response. We thank the reviewer for this incisive question: it led us to quantify rater-level confusion explicitly and to add the result both to Methods and to Limitations. The short answer is yes—the psychiatrists themselves disagree most often on the Moderate/Unwell boundary, which places an empirical upper bound on per-class F1 for classes 1 and 2.

On the canonical 1,768-clip OOF set (Methods, *Assessments and psychiatrist consensus labeling*), we recomputed inter-rater agreement across the three psychiatrists (R1, R2, R3; anonymous identifiers):

- **Fleiss' κ** across all three raters: 0.693 (substantial agreement).
- **Pairwise Cohen's κ**: 0.642 (R1–R2), 0.819 (R1–R3), 0.617 (R2–R3); raw pairwise agreement 81.5% / 90.5% / 80.0%.
- **At least one rater disagreed on 425 / 1,768 clips (24.0%)**; of these, **206 clips (11.6% of the analysis set)** carried both a "Moderate (1)" and an "Unwell (2)" label across the three psychiatrists.

The pairwise confusion matrices (S3 Fig.) confirm that the dominant share of inter-rater disagreement is concentrated on the Moderate/Unwell boundary.

Per-class F1 on the revised Attention-MIL+CORN+LA model (S4 Table) is correspondingly: Healthy = 0.926 (support 1,130; 95% CI $[0.886, 0.956]$), Moderate = 0.704 (support 416; $[0.619, 0.782]$), Unwell = 0.687 (support 222; $[0.474, 0.802]$). We now state explicitly in Discussion, *Limitations and future directions*, that per-class F1 on classes 1 and 2 is upper-bounded by rater-level uncertainty rather than by purely modelling factors, and we point to soft-label training, adjacent-class smoothing, margin-based separation, two-stage hybrid training, and rater-specific heads as natural next steps; the code repository ships pre-staged hooks for the soft-label and ordinal-loss variants.

Reviewer comment: *The use of nonverbal cues has a modest improvement. Maybe it is better not to use it. They increase the computational cost and burden on people. This approach can also improve engagement.*

Response. We agree this is a central question, and the reviewer's challenge prompted us to re-evaluate the evidence under a more rigorous statistical framework.

We agree that multimodal features introduce non-trivial computational costs, and our revised analysis does not support a claim that multimodal features are always necessary or superior to text-only assessment. The revised results suggest that nonverbal modalities may provide additional cues when linguistic information is sparse or ambiguous.

We restructured the modality-ablation analysis around fold-level paired t-tests on the 25 GroupKFold folds, with Holm–Bonferroni correction over the six contrasts against the Text-only baseline (Methods, *Model and evaluation*; Results, *Modality ablation*; Table 2; Table 3).

At the aggregate level the result is dichotomous and does *not* indicate a broad aggregate multimodal advantage:

- **Full vs. Text-only macro-F1 $\Delta = +0.0005$, raw $p = 0.910$, $p_{\mathbf{adj}} = 0.910$** (Holm–Bonferroni); the two configurations are statistically indistinguishable.
- All four text-containing configurations (Full, Audio+Text, Face+Text, Text-only) are statistically indistinguishable from one another on macro-F1, Unwell recall, and ordinal MAE (all $p_{\text{adj}} > 0.40$ in pairwise contrasts).
- All three text-free configurations (Audio+Face, Audio-only, Face-only) are substantially worse than Full ($\Delta < -0.34$, $p_{\text{adj}} < 0.001$).
- Full and Text-only agree on **1,703 / 1,768 OOF clips (96.3%)**; disagreements are concentrated between adjacent severity levels (S1 Table).

Under the re-anchored Holm–Bonferroni analysis, the earlier point estimate of an aggregate Full-vs-Text-only advantage ($\Delta = +0.007$, $p = 0.046$ on a different evaluation set) is no longer statistically significant.

What does survive, and what partially supports the multimodal hypothesis, is an exploratory subgroup contrast (Results, *Subgroup analysis: where nonverbal modalities contribute*). Stratifying by speech-duration tertile and clinical label, in the clinically ambiguous subgroup—short-speech clips with non-Healthy labels ($n = 77$)—Audio+Text significantly outperforms Text-only on macro-F1 (0.478 vs. 0.449, $\Delta = +0.030$, $t_{17} = 2.34$, $p = 0.032$ uncorrected; 18/25 folds analysed under a pre-specified small-stratum exclusion criterion; sensitivity analysis filling excluded folds with $\Delta = 0$ yields $t_{24} = 2.26$, $p = 0.033$; Discussion, *Modality roles and interpretation of nonverbal signals*). We label this finding as exploratory and uncorrected and discuss its multiple-testing caveat in *Statistical multiplicity and the post-hoc nature of subgroup contrasts* (Discussion). In long-speech non-Healthy clips, no configuration significantly outperforms Text-only; in short-speech Healthy clips, all four configurations approach ceiling.

The reviewer's substantive point about cost and engagement is taken up in a new paragraph in Discussion, *Modality roles and interpretation of nonverbal signals* (subparagraph *Deployment tradeoff: when is multimodal worth the additional burden?*), where we explicitly contrast the deployment requirements of Text-only (on-device speech-to-text only) versus Full (camera on, OpenFace pipeline, Parselmouth pipeline). We note that the additional burden of multimodal capture is justified specifically in low-information contexts—short responses from participants whose clinical state is uncertain—rather than as a uniform always-on requirement, although prospective validation of any such deployment strategy is required before clinical translation.

Reviewer comment: *Some text improvements, such as: To address this disconnect.*

Response. We have polished the prose throughout the manuscript, including the specific phrase the reviewer flagged. A native-English copy-editing pass was made on the Introduction, Methods, Results, and Discussion. Edits are visible in the tracked-changes file.

Reviewer comment: *Some explanations are repetitive throughout the text.*

Response. We have tightened the manuscript to remove the redundancies the reviewer identified. The most significant condensations are in the Introduction–Methods transition (presenteeism background no longer restated in the Methods preamble) and in the Discussion (the relation-to-existing-assessments and modality-roles subsections were merged where they previously duplicated material). We additionally removed a stand-alone subsection on Text-only vs. Full disagreement and folded its summary statistic into the Modality-ablation paragraph (the detailed breakdown is retained in S1 Table panel C). Substantive content is preserved.

Reviewer comment: *A better explanation of Figure 3.*

Response. Figure 3 (the reliability diagram, `fig:calibration`) has been rewritten with a four-part self-contained caption: (a) what the figure shows—one-vs-rest reliability diagram aggregated across the three severity classes, computed on OOF clip-level winning-class probabilities (n = 1,768 clips from 29 participants); (b) the protocol—25-fold repeated participant-disjoint cross-validation, equal-width bin partition into 10 bins, observed positive rate plotted against mean predicted probability per bin; (c) the meaning of the symbols and reference line ($y = x =$ perfect calibration); and (d) summary statistics, including the overall ECE of 0.024 over the full probability range. Class-conditional reliability diagrams are integrated into Fig. 3 of the main manuscript (which shows per-class reliability across all four text-containing configurations).

Reviewer #2

Reviewer comment: *Dataset size is very small (only 38 participants), so model may overfit and results may not generalize properly. Authors should show more stability analysis like variance across folds or learning curves.*

Response. We agree, and have substantially deepened the stability analysis.

1. **Evaluation protocol** (Methods, *Model and evaluation*): the evaluation is now 25-fold repeated participant-disjoint cross-validation (5 repeats $\times$ 5-fold GroupKFold). The participant cohort flows from 38 valid video submissions through 34 with three-modality feature extraction and psychiatrist labels (4 participants lacked successful automatic transcription on all clips and were not part of the analysis set; Methods, *Feature extraction and preprocessing*) to 29 after the participant-level short-span exclusion criterion ($\leq$ 14 days

of recording activity, ≈ 0.7% of clips), yielding the canonical analysis set of **1,768 OOF clips from 29 participants**. Fold-level variance is reported directly across all 25 folds.

2. **Cluster bootstrap** (Methods, *Model and evaluation*): a participant-level cluster bootstrap with $B = 2{,}000$ **resamples** of the 29 participants now produces 95% percentile CIs for every headline metric. The participant level is the appropriate unit of inference because the GroupKFold splits are participant-disjoint.
3. **Reported intervals** (Table 1): macro-F1 = 0.772, CI $[0.697, 0.817]$; accuracy = 0.840, CI $[0.796, 0.883]$; macro-AUROC = 0.940, CI $[0.915, 0.958]$; macro-AUPRC = 0.800, CI $[0.715, 0.846]$; overall ECE = 0.024. Per-class F1 CIs are in S4 Table, and the participant-level bootstrap distributions for overall and per-class metrics are shown in S2 Fig.

Wider intervals on the Moderate and Unwell classes mirror their smaller support (416 and 222 clips) and the rater-level ambiguity quantified in our response to R1-1; the bootstrap protocol exposes that uncertainty rather than hiding it.

Reviewer comment: *Strong class imbalance, especially "unwell" class is very less; using class-weighted loss or focal loss can improve this.*

Response. We agree that class imbalance is a primary concern, and the revised pipeline addresses it through two complementary mechanisms (Methods, *Model and evaluation*):

1. **CORN ordinal head** on the fused logits, which exploits the rank structure of the severity scale and softens the boundary between adjacent severity levels in the loss.
2. **Logit adjustment** (LA, $\tau = 1.0$) in the fusion step, which corrects the decision boundary for the empirical class prior without modifying the loss function itself.

A small CORAL-style auxiliary BCE on the text-stream output ($\lambda_{\text{aux}} = 0.2$) provides additional ordinal regularisation.

The decision to use CORN+LA is supported by a preliminary loss-function sensitivity sweep on an earlier (Transformer-based) architecture, now reported transparently in **S3 Table** (Methods discussion in Results, the opening of *Limitations and future directions* (loss-sensitivity paragraph) paragraph):

Variant (earlier architecture; single split)	macro-F1	F1 (mod.)	F1 (unwell)
Cross-entropy (baseline)	0.607	0.612	0.353
Weighted CE	0.618	0.575	0.414
Focal ($\gamma = 2$)	0.618	—	0.429
CORN (ordinal)	0.609	0.631	0.357
**CORAL (ordinal)**	**0.629**	**0.661**	0.355
Soft-KL (rater mean)	0.481	0.445	0.219

CORAL is the overall winner of the sweep; CORN (its rank-consistent variant adopted here) provided the best Moderate-class F1 within the family. Weighted CE and focal each improved F1 on the rare Unwell class but did not surpass the ordinal family on macro-F1. *Caveat*: the absolute values in S3 Table are from a different (earlier) architecture and a single fixed split; they are reported solely as ablative evidence for the choice of CORN+LA in the Attention-MIL model and are not comparable to the headline OOF results.

Under the revised Attention-MIL+CORN+LA model on the 1,768-clip OOF set (S4 Table), Unwell-class F1 = 0.687 (recall 0.667) and Moderate-class F1 = 0.704; this is the regime against which all claims in the paper are now made.

Reviewer comment: *Moderate class is getting confused more, which shows ordinal modeling is not fully effective; better ordinal loss or margin-based separation can be used.*

Response. We agree and have adopted the rank-consistent ordinal family in the revised model. The primary training objective is the **CORN ordinal loss**, which encodes the cumulative constraint that class 2 $\Rightarrow$ class 1 $\Rightarrow \neg$ class 0 through a chain of binary classifiers (Methods, *Model and evaluation*). CORN is combined with logit adjustment (R2-2) for class-prior correction and with a small CORAL-style BCE auxiliary loss on the text stream for additional ordinal regularisation.

On the revised 25-fold OOF predictions (S4 Table; confusion matrices in S7 Table):

- Moderate-class F1 = 0.704 (95% CI $[0.619, 0.782]$); recall = 0.762; precision = 0.654.
- Unwell-class F1 = 0.687 (95% CI $[0.474, 0.802]$); recall = 0.667; precision = 0.708.
- Off-diagonal confusion remains concentrated on the adjacent Moderate/Unwell boundary; direct Healthy$\leftrightarrow$Unwell confusions are rare (4 + 11 of 1,768).

The reviewer's suggestion of **margin-based separation** is now explicitly flagged in Discussion, *Limitations and future directions*, as a natural next step alongside adjacent-class smoothing, two-stage hybrid (hard$\rightarrow$soft) training, and rater-specific heads. We have not implemented a full margin-based ordinal regression in this revision because the rater-level upper bound on classes 1/2 (R1-1) suggests that the remaining error budget is partly irreducible without changes to the labelling protocol; we therefore prefer to flag margin methods as a future direction rather than to advertise a marginal numerical improvement.

Reviewer comment: *Multimodal fusion — results show text is dominating and other modalities are not contributing much; fusion strategy needs improvement.*

Response. The reviewer's observation is correct on the aggregate, and we now report it explicitly. The detailed treatment is in our response to R1-2 above; we summarise here.

Aggregate level (Results, *Modality ablation*; Table 2): under the late-fusion Attention-MIL with Holm–Bonferroni–corrected paired contrasts (six contrasts vs. Text-only), Full is statistically *indistinguishable* from Text-only ($\Delta = +0.0005$, $p_{\text{adj}} = 0.910$); Audio+Text and Face+Text are likewise indistinguishable from Text-only ($\Delta = -0.0049$, $p_{\text{adj}} = 0.527$; $\Delta = -0.0054$, $p_{\text{adj}} = 0.494$, respectively). Text-free configurations are substantially worse ($\Delta < -0.33$, $p_{\text{adj}} < 0.001$). The text stream is necessary; the nonverbal streams are not uniformly additive on top of it.

Subgroup level (Results, *Subgroup analysis: where nonverbal modalities contribute*): in the short-speech $\times$ non-Healthy stratum ($n = 77$)—the clinically ambiguous subgroup—Audio+Text significantly outperforms Text-only ($\Delta = +0.030$, $t_{17} = 2.34$, $p = 0.032$ uncorrected, exploratory). No other subgroup contrast reaches significance.

What this implies for fusion strategy. Late fusion at the logit level, with stream-level attention weights, is sufficient to preserve the Text-only signal without degradation and to recover a statistically detectable acoustic complement in the clinically ambiguous subgroup. We do not claim that a more complex fusion strategy could not do better; we claim that the empirical case for the multimodal hypothesis rests on (a) the necessity of text (text-free configurations perform substantially worse with $\Delta < -0.33$) and (b) selective acoustic superiority in the clinically ambiguous subgroup ($p = 0.032$ in short-speech $\times$ non-Healthy)—not on aggregate dominance. We have rewritten Discussion, *Modality roles and interpretation of nonverbal signals*, to make this argument explicit, and added an LLM-driven adaptive-prompt direction in *Limitations and future directions* as a way to elicit richer affective dynamics that might allow the face stream to contribute beyond its current marginal role.

Reviewer comment: *Evaluation part needs more depth, only average metrics are given, but confidence intervals or statistical significance across folds are not shown.*

Response. Three additions address this comment.

1. **Cluster-bootstrap 95% CIs** on every reported metric (Methods, *Model and evaluation*; Table 1 and S4 Table; S2 Fig.). The bootstrap resamples participants ($B = 2{,}000$ resamples of $n = 29$), which is the appropriate unit of inference given the participant-disjoint splits.
2. **Within-protocol paired t-tests** across the 25 folds for every modality configuration against Full (Methods, *Model and evaluation*; Table 2; Table 3). Each fold is held fixed across configurations so that fold variance does not contaminate the contrast.
3. **Holm–Bonferroni correction** across the family of six paired contrasts against the Text-only baseline, which controls the family-wise error rate at the modality-ablation level. We additionally added a dedicated subsection *Statistical multiplicity and the post-hoc nature of subgroup contrasts* (Discussion) that flags the subgroup contrast in *Subgroup analysis: where nonverbal modalities contribute* as exploratory and uncorrected.

Fold-mean standard deviations are reported directly in Table 2 (e.g., Full macro-F1 fold SD = 0.076 across 25 folds).

Reviewer comment: *Calibration is reported but only overall ECE, class-wise calibration is missing.*

Response. Per-class (one-vs-rest) calibration is now reported alongside the overall ECE in Results, *Discrimination and calibration analyses*:

Class (one-vs-rest)	ECE	Brier	Support
0 (healthy)	0.038	0.067	1,130
1 (moderate)	0.024	0.102	416
2 (unwell)	0.025	0.059	222

Overall ECE = 0.024. Per-class ECE is small and similar across classes (≤ 0.04). The Moderate class carries the largest *Brier* score (0.102), consistent with the rater-level ambiguity quantified in our response to R1-1, even though its binned ECE is small. The accompanying per-class reliability diagrams are shown directly in **Fig. 3 of the main manuscript** (per-class panels across all four text-containing configurations), with the same binning protocol (10 equal-width bins on $[0, 1]$).

Reviewer comment: *Labels depend on psychiatrist agreement (kappa 0.685), so there is some subjectivity, model should handle label uncertainty more properly using soft labels.*

Response. We agree that label uncertainty warrants explicit treatment, and we implemented a soft-label variant using KL-divergence against the rater-mean soft target (the empirical (p_0, p_1, p_2) obtained by averaging the three psychiatrists' one-hot vectors). We report this transparently, including the negative result: in our preliminary single-stage sweep on the earlier architecture (S3 Table), **soft-KL substantially underperformed cross-entropy on macro-F1 (**0.481 **vs.** 0.607**)**, and degraded every per-class F1 (Healthy 0.855 → 0.777; Moderate 0.612 → 0.445; Unwell 0.353 → 0.219) and ordinal MAE (0.281 → 0.430).

The plausible explanation, given in Discussion, *Limitations and future directions*: the soft target has low sharpness (Fleiss $\kappa = 0.693$; mean per-clip entropy near one bit), and used as the *sole* training objective the KL signal under-trains the classification head. We therefore did not adopt single-stage soft-label training for the main Attention-MIL model. Instead, the revised pipeline uses CORN ordinal training plus logit adjustment, which provides an implicit form of ambiguity-aware learning by softening the loss-side boundary between adjacent severity levels rather than the target-side distribution.

A **two-stage hybrid** (hard-label warm-up followed by soft-label fine-tuning), **ambiguity-aware variants**, and **rater-specific heads** are flagged as immediate future directions in *Limitations and future directions*; the code repository ships pre-staged hooks for the soft-label and ordinal-loss extensions to facilitate this work. We have updated the inter-rater κ value: the original submission reported $\kappa = 0.685$, which was the value on an earlier label set; the canonical 1,768-clip analysis set yields Fleiss $\kappa = 0.693$ (pairwise Cohen κ 0.642 / 0.819 / 0.617), which we now use consistently.

Reviewer comment: *Feature extraction depends on external tools like OpenFace and Whisper, so errors from these tools can affect results, but this is not discussed clearly.*

Response. We have added a dedicated paragraph on upstream tool-error propagation in Discussion, *Limitations and future directions*, structured around the three stages at which we mitigate tool-induced noise:

1. **Data-curation stage.** Clips with an OpenFace tracking-success ratio below 0.20 or a Parselmouth voiced-frame ratio below the configured floor were removed, and clips for which any of the three modality CSVs failed to be produced were excluded entirely. The 1,768 OOF clips reported in the manuscript all passed every quality gate.
2. **Modelling stage.** Per-window blended normalisation (per-user $\times$ per-cohort z-scoring with a Bayesian shrinkage prior) suppresses systematic shifts characteristic of stable tool error modes, e.g., a miscalibrated F0 floor for a specific recording session or a persistent gaze-tracking offset for a specific user.
3. **Evaluation stage.** The modality ablation (Table 2) shows that removing *both* nonverbal modalities (the Text-only configuration) yields macro-F1 that is statistically indistinguishable from the best fused configuration within the same protocol ($\Delta = +0.0005$, $p_{\text{adj}} = 0.910$). This provides an *empirical upper bound* on the worst-case impact of OpenFace and Parselmouth tool errors on headline performance: if both tools were producing pure noise, headline macro-F1 could at most fall back to the Text-only baseline, which it does not.

Whisper errors, in contrast to OpenFace and Parselmouth, would affect the dominant text channel and are *not* bounded by the modality-ablation argument; we acknowledge this openly and flag manual spot-checking of a stratified sample and comparison against alternative ASR backends as a useful direction for future validation work.

Summary of new content in the revised manuscript

We are grateful to the reviewers for guidance that materially improved both the rigour and the transparency of the paper. The most important consequence of the revision is that the empirical claim about multimodal fusion is now aligned with the actual data: the multimodal hypothesis is partially supported by the necessity of text (text-free configurations perform substantially worse) and selective acoustic superiority in the clinically ambiguous subgroup ($p = 0.032$ in short-speech $\times$ non-Healthy)—not aggregate dominance. We hope that this re-anchored, transparently reported version of the paper addresses every point to the reviewers' and editor's satisfaction.

Section	What was added or changed	Item
Model and evaluation	Transformer → Attention-MIL (3-stream, late logit fusion, CORN ordinal head, logit adjustment, CORAL text-side auxiliary)	R2-2, R2-3, R2-4
Evaluation protocol	Re-anchored on 25-fold repeated GroupKFold OOF (n = 1,768, 29 participants); cluster bootstrap ($B = 2{,}000$) for CIs; Holm–Bonferroni for modality contrasts	R2-1, R2-5
Methods → *Assessments ...*	Updated Fleiss' κ = 0.693; pairwise Cohen κ 0.642/0.819/0.617; 425/1,768 = 24.0% inter-rater disagreement; 11.6% Moderate/Unwell co-labelling	R1-1, R2-7
Results → *Overall performance*	Cluster-bootstrap 95% CIs on every headline metric; per-class CIs in S4 Table	R2-1, R2-5
Results → *Discrimination and calibration*	Per-class ECE and Brier with class-wise interpretation; per-class reliability diagrams integrated into Fig. 3 of the main manuscript	R2-6
Results → *Modality ablation*	25-fold paired-t contrasts against Text-only baseline with Holm–Bonferroni; 4 text-containing configs presented in parallel (no privileged reference); 3 text-free configs as control; clip-level agreement statistic	R1-2, R2-4
Results → *Subgroup analysis: where nonverbal modalities contribute*	Exploratory subgroup analysis; significant short × non-Healthy contrast ($n = 77$, $p = 0.032$ uncorrected)	R1-2, R2-4
Discussion → opening of *Limitations and future directions* (loss-sensitivity paragraph)	Honest reporting of S3 Table sweep; soft-KL negative result; CORN+LA choice rationale	R2-2, R2-3, R2-7
Discussion → *Modality roles and interpretation of nonverbal signals*	Non-inferiority + subgroup-superiority rationale; deployment-tradeoff discussion contrasting Text-only vs. Full burden; LLM-prompt future direction	R1-2, R2-4
Discussion → *Statistical multiplicity...*	Explicit multiplicity caveat for the subgroup contrast; replication / hierarchical-FWE flagged as required	R2-5
Discussion → *Limitations and future directions*	(a) rater upper bound on per-class F1; (b) margin-based separation as future work; (c) two-stage hybrid soft-label training; (d) 3-stage tool-error mitigation argument	R1-1, R2-3, R2-7, R2-8
Supporting Information (restructured per R0-7)	3 figures (S1 PR / S2 bootstrap / S3 rater confusion) + 7 tables (S1 Text-only/Full agreement / S2 hyper-parameter search / S3 loss sweep / S4 per-class metrics with CIs / S5 baseline questionnaire / S6 daily EMA / S7 confusion matrices of four text-containing configurations)	R0-7, R2-2, R2-6, R2-7
